# High-Reliability Thermoreceptors with Minimal Temporal and Spatial Variations Through Photo-Induced Patterning Thermoelectrics

**DOI:** 10.1007/s40820-025-01821-1

**Published:** 2025-06-23

**Authors:** Chunyu Du, Yue Hu, Xiao Xiao, Farid Manshaii, Lirong Liang, Jun Chen, Guangming Chen

**Affiliations:** 1 College of Materials Science and Engineering, Shenzhen University, Shenzhen, 518055 People’s Republic of China; 2 Department of Bioengineering, University of California, Los Angeles, CA 90095 USA; 3 SKKU Institute of Energy Science and Technology, Sungkyunkwan University, Suwon, 16419 Republic of Korea

**Keywords:** Thermoelectric composites, Wearable electronics, Artificial thermoreceptors, Sensors

## Abstract

The development of bionic sensing devices with advanced physiological functionalities has attracted significant attention in flexible electronics. In this study, we innovatively develop an air-stable photo-induced *n*-type dopant and a sophisticated photo-induced patterning technology to construct high-resolution joint-free *p*–*n* integrated thermoelectric devices. The exceptional stability of the photo-induced *n*-type dopant, combined with our meticulously engineered joint-free device architecture, results in extremely low temporal and spatial variations. These minimized variations, coupled with superior linearity, position our devices as viable candidates for artificial thermoreceptors capable of sensing external thermal noxious stimuli. By integrating them into a robotic arm with a pain perception system, we demonstrate accurate pain responses to external thermal stimuli. The system accurately discerns pain levels and initiates appropriate protective actions across varying intensities. Our findings present a novel strategy for constructing high-resolution thermoelectric sensing devices toward precise biomimetic thermoreceptors.

**Supplementary Information:**

The online version contains supplementary material available at 10.1007/s40820-025-01821-1.

## Highlights

- A novel photobase generator is specifically designed for the fabrication of high-resolution sensing devices.
- Similarities in pain perception mechanism between thermoelectric-based artificial thermoreceptor and biological nociceptor.
- Emulation common nociceptive behaviors and pain response under excessive temperature stimuli.

**Supplementary Information:**

The online version contains supplementary material available at 10.1007/s40820-025-01821-1.

## Graphical abstract

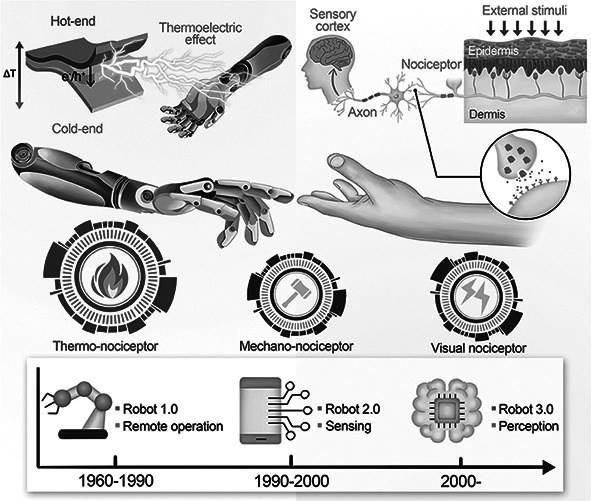

## Introduction

The rapid development of bionic intelligent sensing technology, combined with the continuous advancement of information technology such as artificial intelligence and big data, has facilitated important advancements in functional materials, flexible electronics, and humanoid robotics [1–7]. These developments are driven by interest in bionic sensors, which can be classified into visual, auditory, and tactile types based on their unique structures and working mechanisms [8–12]. Each type is tailored to adapt to specific functions within different scenarios [13–18]. However, more sophisticated physiological functions, such as the human-like injury responses, remain underdeveloped [19]. For instance, when individuals are exposed to noxious stimuli from the external environment, a series of complex physiological responses are triggered. These responses include a sense of pain, visible signs of injury, and healing processes, all designed to protect the individual from harm or further damage. Consequently, there is significant interest and demand for advanced electronics that integrate perception capabilities.

Pain perception is central to the body’s physiological protection mechanisms. The detection of physical pain is achieved through nociceptors, a type of sensory receptor that encodes noxious stimuli into neural signals and sends “possible danger” signals to the spinal cord and brain. This triggers various physiological and behavioral responses [20, 21]. A biological nociceptor is characterized by five distinct features: (i) a “threshold” to trigger pain; (ii) “no adaptation,” meaning it continues to trigger in reaction to recurrent noxious stimuli; (iii) “relaxation” after the noxious stimuli have ceased; and abnormal sensitization, known as (iv) “allodynia” and (v) “hyperalgesia,” while the injury remains. Nociceptors are primarily divided into mechanonociceptors, visual nociceptors, chemical nociceptors, and thermoreceptors [22–25]. Among them, thermoreceptors exhibit bimodal functionality, integrating both thermal sensing and nociceptor function through shared mechanism. Although previous studies have reported on the mechanonociceptive, visual, and chemical nociceptive behaviors of semiconductor devices, including complementary metal oxide semiconductors, transistors, and resistive access memories, thermo-nociceptive system are still in their early stages of development [26–30]. Thus, the pursuit of appropriate functional materials to facilitate the design of optimal thermoreceptors is imperative to address the current void in nociceptor classification.

**Fig. 1:**
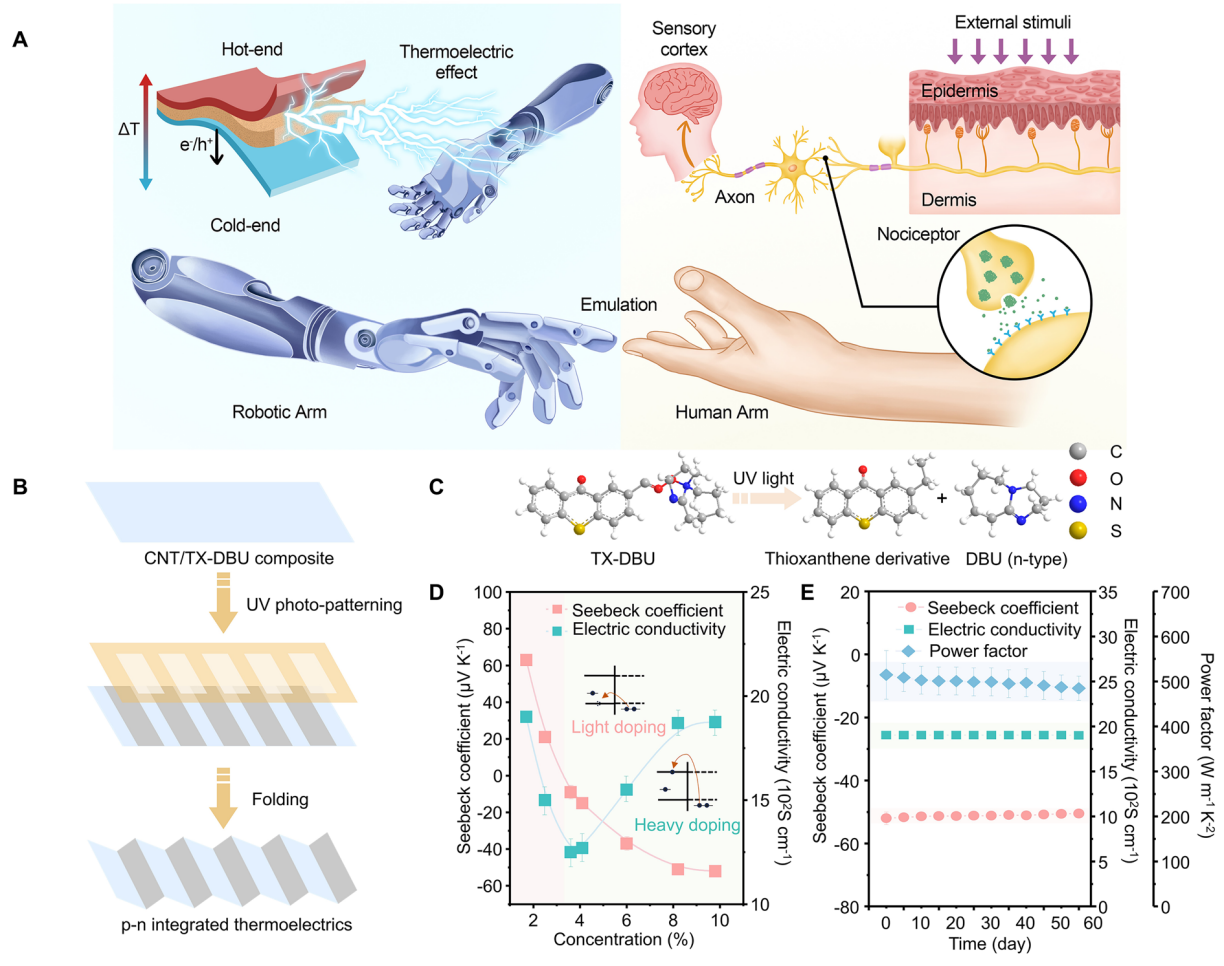
Concept of a robotic arm with an artificial thermoelectric nociceptive system and performance of the as-prepared composite samples. **A** Left: Working principle of the robot featuring an artificial thermoelectric nociceptive system. Right: Structure of a biological nociceptive sensory system. This thermoelectric nociceptive system closely mirrors biological pain perception mechanisms. **B** Schematic diagram of *p*–*n* integrated thermoelectrics using UV-induced patterning with origami techniques. **C** Reaction Schematic Diagram of photobase generator after UV exposure. **D** Thermoelectric performance with the increase in photobase generator concentration. **E** Thermoelectric performance over varying exposure times in air

Flexible thermoelectric devices show great potential for transforming the field of bionic sensing devices [31–38]. These devices not only capitalize on the inherent benefits of thermoelectric materials but also introduce new levels of conformability and mechanical robustness, which exhibit remarkable advantages when serving as artificial thermoreceptors (Note S1). First, their sensing mechanism closely aligns with the biological pain perception mechanism, where both biological nociceptors and thermoelectric-based artificial thermoreceptors activate protective mechanisms by converting harmful external thermal stimuli into electrical signals. Secondly, these devices operate on the principle of the Seebeck effect, exhibiting excellent linearity as the thermoelectric potential linearly correlates with the temperature difference. Third, the device structure, composed of thermoelectric material sandwiched by two electrodes, allows for easy integration. Lastly, these self-powered thermoreceptors can utilize heat sources for direct power supply, thus effectively addressing endurance issues (Figs. 1A and S1).

Despite their many advantages, the application of thermoelectric devices in high-performance bionic sensing faces a common challenge: inherent temporal and spatial variations [39]. Temporal variation refers to changes in device performance over time. Such changes mainly stem from device aging and material stability [40]. Over extended periods of use, continuous temperature stimuli may lead to Joule heating at the junction between thermoelectric and metallic connection materials, resulting in degraded device performance and subsequently impacting its reliability and stability. In contrast to temporal variation, spatial variation pertains to differences in device performance across different spatial locations [41]. These differences may arise from process deviations during device manufacturing and material inhomogeneity. The inhomogeneity at the interface between thermoelectric materials and metallic connecting materials can lead to variations in the thermoelectric performance of the device at different locations. Furthermore, for doped *n*-type thermoelectric materials, the often uneven doping concentration during the doping process poses another issue that contributes to significant spatial variations in thermoelectric device performance. Recent advances in integrated devices through photopatterning techniques, such as laser ablation and oxygen plasma etching, have unlocked new capabilities for precision material processing [42–44]. However, the pursuit of high-performance bionic sensing devices now demands even greater attention to critical parameters, including non-destructive spatial uniformity to ensure consistent thermoelectric properties at sub-micron scales, and long-term interfacial stability to maintain performance integrity over extended thermal cycling. Additionally, material-specific challenges—such as dopant segregation, which can impair carrier mobility, and surface oxidation, which elevates contact resistance—underscore the need for refined strategies to achieve optimal device functionality. Addressing these factors is essential to advancing device toward exceptional reliability and performance standards. Necessitating innovative interface engineering, advanced doping control to meet the rigorous standards of next-generation sensing applications. Therefore, there is an urgent need to develop more advanced material and ingenious device to minimize the variations in thermoelectric devices, thereby fulfilling their application requirements in biomimetic thermoreceptors.

In this study, we have innovatively developed a novel photobase generator, (9-oxo-9H-thioxanthen-2-yl)-acetate-2,3,4,6,7,8,9,10-octahydro-1H-pyrimido[1,2-a]azepin-5-ylium (TX-DBU), capable of producing an organic base with electron-donating properties under UV irradiation, rendering it an exceptional *n*-type dopant for carbon nanotubes (CNTs). Upon UV exposure, this *n*-type dopant exhibits outstanding air stability, preserving its thermoelectric performance in air for a duration exceeding 60 days. Building upon this, we skillfully integrated template patterning with origami techniques to successfully synthesize high-precision *p*–*n* integrated thermoelectric devices. Within these devices, the *p*-type material comprises CNTs, whereas the *n*-type material consists of CNTs doped by the photobase generator post-UV exposure. Notably, the seamless integration of *p*-type and *n*-type materials is achieved through a carbon nanotube network, eliminating the need for any metal connecting materials. Attributable to the exceptional stability of the photobase generator post-UV exposure and our meticulously crafted joint-free, high-precision *p*–*n* integrated thermoelectric device architecture, the device demonstrates remarkably low temporal and spatial variations. Consequently, this thermoelectric device can serve as an artificial thermoreceptor, effectively mimicking crucial nociceptor features such as threshold, non-adaptation, relaxation, allodynia, and hyperalgesia. Ultimately, we successfully integrated these artificial thermoreceptors into a robotic arm, enabling it to generate pain responses upon exposure to excessive external thermal stimuli, thereby demonstrating the precise perception of external harmful stimuli by the biomimetic thermo-nociceptive robotic arm.

## Experimental Section

### Chemicals and Materials

DBU and thioxanthone-carboxylic acid (TX) were purchased from Aladdin. The CNT was purchased from Nanjing XFNANO. All other chemicals utilized in the experiment were of reagent grade and were used without additional purification.

### Synthesis of TX-DBU

The synthesis method for the synthesis of TX-DBU was based on the procedure outlined in the referenced literature [45]. DBU (0.127 g) was introduced to a thioxanthone-carboxylic acid (TX)-Tetrahydrofuran solution (1 mmol) and then stirred thoroughly at room temperature. Subsequently, a yellowish viscous oil was obtained and purified through washing with toluene.

### Fabrications of TX-DBU/CNT Films

Firstly, the CNT film was immersed in a solution of TX-DBU, maintaining a TX-DBU to CNT ratio of approximately 6.3:100. Secondly, a mask plate with an interphase strip distribution pattern was utilized to overlay the TX-DBU/CNT composite surface. Finally, UV light (λ = 365 nm, 100 mW cm⁻^2^) was applied for irradiation to fabricate the TX-DBU/CNT films.

### Fabrications of TX-DBU/CNT *p*–*n* Integrated Thermoelectric Device (Thermoelectric Nociceptor)

The prepared TX-DBU/CNT films were folded into a zigzag shape along the *p*–*n* junction to form a vertical device, with silver paste used to connect electrodes at both ends of the device. The resulting thermoelectric device (thermoelectric nociceptor) was subsequently encapsulated with polyimide films.

### Fabrication of Biomimetic Thermo-Nociceptive Robotic Arm

The thermo-nociceptive biomimetic robotic arm consists of two primary components: a nociceptor system and a pain response system. The former encompasses the thermoelectric nociceptor, a voltage amplifier, and a voltage control module. The latter is a robotic arm, compatible with Arduino programming and STM32, featuring five fingers propelled by five anti-locked LFD-01 servo motors. The thermoelectric nociceptor is linked to a voltage amplifier to magnify the electrical voltage generated by the thermoelectric sensing elements. The amplified electric voltage is subsequently conveyed to the voltage control module to activate the servo motor. The STM32 microcontroller serves to convert the produced input analog signal into a digital signal. Moreover, the Bluetooth module is connected to the STM32 microcontroller to preset the hand motion.

### Characterization and Measurement

The sample morphology was analyzed utilizing a scanning electron microscope (SEM, Apreo S, FEI). Raman spectra were recorded using a Raman spectrometer (XploRA plus, Horiba) operating in regular mode with a 532 nm excitation wavelength. X-ray photoelectron spectroscopy (XPS) was conducted using a surface analysis system (5000C ESCA, PHI) equipped with an Al Ka anode, with a power of 100 W. The Seebeck coefficient and electrical conductivity of the samples were evaluated using a thin film thermoelectric parameter test system (MRS-3RT, Wuhan Joule Yacht) operating in a quasi-steady-state mode. Prior to measurement, the samples were sectioned into rectangular shapes with dimensions of approximately 30 mm in length and 10 mm in width. The simulation capabilities of our thermoelectric nociceptor were conducted utilizing a customized thermoelectric testing system comprising a smart thermostat (719P/K5, Udian) and a digital multimeter (2700 Keithley).

## Results and Discussion

### Fabrication and Performance of Photo-Induced Patterning Thermoelectrics

Photo-induced patterning with origami techniques was applied to fabricate high-precision *p*–*n* integrated thermoelectric devices (Fig. 1B). Initially, a uniform coating of yellow TX-DBU was applied onto a thin film composed of CNTs. Due to the oily liquid properties of TX-DBU, it achieved thorough and homogeneous composite formation on the CNT surface, thereby mitigating potential material heterogeneity concerns stemming from uneven distribution. Subsequently, a mask plate featuring an interphase strip distribution pattern was employed to overlay the TX-DBU/CNT composite surface. Upon exposure to ultraviolet radiation (*λ*_ex_ = 365 nm, 50 mW cm^−2^), TX-DBU underwent decarboxylation, resulting in the liberation of 1,8-diazabicyclo[5.4.0]undec-7-ene (DBU) molecules (Fig. 1C). Notably, DBU, an organic base exhibiting substantial electron-donating capabilities, effectively facilitates the transition of CNTs from their intrinsic state to *n*-type semiconductors when doped. Importantly, UV light selectively induces the *n*-type conversion of CNTs solely within the open regions of the mask plate, while the TX-DBU/CNTs shielded by the mask retain their original *p*-type characteristics. This methodology not only enables high-precision control during the material modification process but also successfully fabricates structures characterized by extremely narrow *p*–*n* transition zones, paving the way for the development of high-precision *p*–*n* integrated thermoelectric devices (Fig. S2). Finally, an innovative Z-shaped vertical high-precision *p*–*n* integrated thermoelectric device structure was constructed. This structure is designed to align the *p*-type and *n*-type regions in a face-to-face configuration, ensuring direct contact between the *p*- and *n*-domains. The alignment and contact are achieved through the application of origami technology, which allows for precise and efficient assembly of the thermoelectric device. To evaluate the *n*-type doping effect and environmental stability of TX-DBU following photo-induction, a series of experiments were conducted. As illustrated in Fig. 1D, with the gradual increase in the concentration of UV-irradiated TX-DBU dopant, the Seebeck coefficient of the UV-irradiated TX-DBU/CNT composite undergoes a transition from *p*-type (initially at 62 μV K^−1^) to *n*-type, reaching a minimum of − 45 μV K^−1^, and subsequently remains relatively constant as the dopant concentration further increases. At higher concentrations of TX-DBU, the carrier concentration approaches the effective density of states within the material. Given the logarithmic relationship between the Seebeck coefficient and the carrier concentration, the rate of change in carrier concentration significantly decreases as it nears the saturation threshold of the density of states. Consequently, the Seebeck coefficient stabilizes, showing little variation despite further increases in the dopant concentration. Notably, the *p*–*n* transition in the UV-irradiated TX-DBU/CNT composite occurs at a dopant concentration of approximately 3.5%. The electrical conductivity of the UV-irradiated TX-DBU/CNT composite exhibits a trend of initial decrease followed by increase with the increasing concentration of UV-irradiated TX-DBU dopant. Specifically, the conductivity decreases from 1870 to 1250 S cm^−1^ and then rises back to 1820 S cm^–1^. This behavior can be attributed to the fact that at low doping levels, electrons from the UV-irradiated TX-DBU dopant neutralize holes in the CNTs, leading to a reduction in both Seebeck coefficient and electrical conductivity. However, under heavy doping conditions, the CNTs become dominated by electrons donated by UV-irradiated TX-DBU, resulting in an increase in both Seebeck coefficient and electrical conductivity. To investigate the air stability of TX-DBU/CNT composites after UV irradiation, we continuously monitored their Seebeck coefficient and electrical conductivity over a period of 60 days. As illustrated in Fig. 1E, the air stability of both the Seebeck coefficient and electrical conductivity within the *n*-type region of the UV-irradiated TX-DBU/CNT composites remained essentially stable for 60 days. The power factor, after 60 days of air exposure, retained more than 95% of its initial value. The stability of most *n*-type dopants is often compromised due to two primary factors: air-based oxidation that converts *n*-type dopants, leading to reduced stability, and the production of oxygen radicals with water, which hinders their electron-donating capacity [46]. Despite the strong water-absorbing properties of DBU, the UV-irradiated TX-DBU/CNT composites exhibited good air stability. This stability can be attributed to two factors: the formation of a compact, light yellow TX film on the surface of the CNT composite following TX-DBU incorporation, which serves as a protective barrier against further oxidation by oxygen and water molecules in the air; and the markedly enhanced electron-donating ability of TX-DBU after UV irradiation, which effectively counteracts the detrimental effects imparted by oxygen radicals generated from water interactions on its electron-donating functionality.

**Fig. 2:**
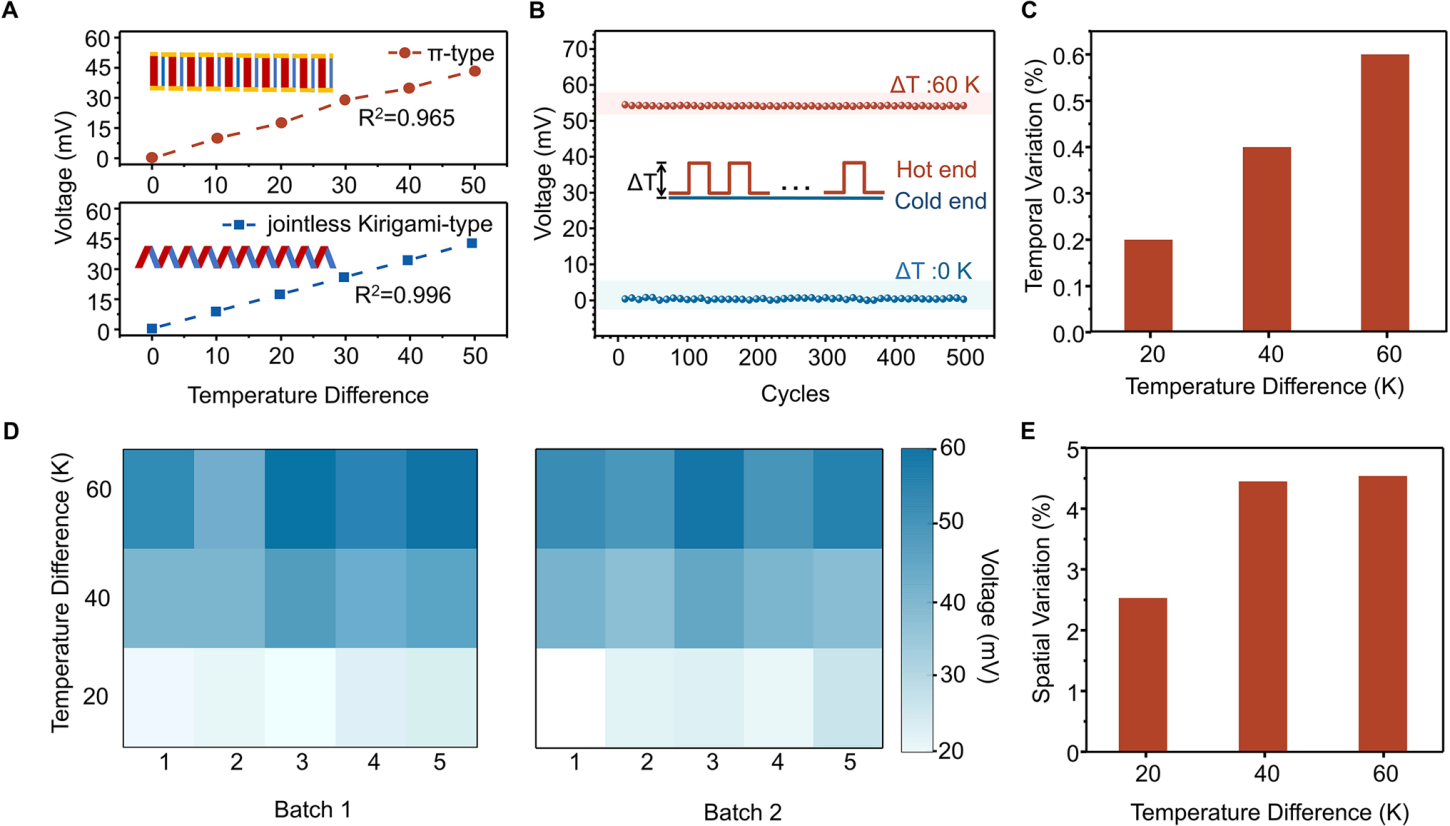
Performance of thermoelectric devices. **A** Open-circuit voltages as a function of temperature differences of π-type thermoelectric device and *p*–*n* integrated thermoelectric device; inset: Schematic illustration of 10 *p*–*n* paired π-type thermoelectric device and 10 *p*–*n* paired *p*–*n* integrated thermoelectric device. **B** Temporal voltage variation with a temperature difference cycle ranging from 0 and 60 K. **C** Temporal voltage variation of the thermoelectric device with temperature differences at 20, 40, and 60 K. **D** Map of the spatial variation test. **E** Spatial variation of thermoelectric device voltage with temperature differences at 20, 40, and 60 K

Given the uniformity and stability exhibited by UV-irradiated TX-DBU/CNT composites, coupled with advancements in device structure refinement and the implementation of a joint-free design, TX-DBU/CNT *p*–*n* integrated thermoelectric devices exhibit remarkable reproducibility and stability. The voltage–temperature curves for the TX-DBU/CNT *p*–*n* integrated thermoelectric device and the multi-leg series-type thermoelectric device are depicted in Fig. 2A. Compared to the multi-leg series-type thermoelectric device, the TX-DBU/CNT *p*–*n* integrated thermoelectric device exhibits superior linearity in voltage with respect to temperature, with an *R*^*2*^ value of 0.996. The primary cause of fluctuation in multi-leg series-type thermoelectric devices is the Schottky contact, which arises when a metal comes into contact with a semiconductor material, forming an electrical barrier at the interface known as the Schottky barrier. This barrier controls the flow of electrons, enabling current rectification and modulation [47]. In multi-leg series-type thermoelectric devices, *p*-type thermoelectric materials are connected with *n*-type thermoelectric materials through metallic connections such as conductive silver paste or copper foil. The formation of Schottky contacts between thermoelectric materials and metallic connection materials adversely affects the high-precision sensing performance of thermoelectric devices. Furthermore, the interface between metallic connection materials and thermoelectric materials can introduce parasitic thermal resistance, which interferes with accurate temperature measurement at both the hot and cold ends, thereby reducing the accuracy and reliability of the biomimetic thermoelectric sensor. Conversely, the TX-DBU/CNT *p*–*n* integrated thermoelectric device avoids Schottky contacts and parasitic thermal resistance-induced voltage–temperature linearity degradation due to the absence of metallic connectors and the utilization of a carbon nanotube network throughout the device.

As shown in Fig. 2B, TX-DBU/CNT *p*–*n* integrated thermoelectric device consistently produces the same output voltage under an identical temperature difference, demonstrating exceptional temporal reliability under temperature stimuli. After enduring 500 temperature cycles, the device shows robust reproducibility. The temporal variation, represented as *σ/μ* (where *μ* denotes the mean voltage and *σ* represents the standard deviation), is evaluated by measuring the output voltage. As shown in Fig. 2C, the temporal variation of the thermoelectric device was less than 1% across a temperature difference range from 0 to 60 K, which shows a dramatic reduction compared with traditional thermoelectric devices (Fig. S3). This low temporal variation can be attributed to the joint-free device structure, which eliminates temporal variations caused by Joule heating at the junction between thermoelectric and metallic connection materials under continuous temperature stimuli (Fig. S4). Furthermore, the integrated devices show excellent temporal reliability across repeated bending cycles, as evidenced by their stable thermoelectric performance under mechanical deformation (Fig. S5). This remarkable durability underscores their potential for practical wearable applications requiring both flexibility and reliable energy conversion capabilities.

In addition to its exceptional temporal reliability and stability, the device also shows significant uniformity in the spatial variation of the output voltage. This variation is evaluated by analyzing thermoelectric devices from two different batches, where both batches utilized identical materials, processing protocols, and manufacturing conditions. The only distinction between the batches was their temporal separation during production, ensuring that any observed differences in spatial voltage variation arise solely from inherent device-to-device variability (Fig. 2D). Figure 2E illustrates the spatial distribution of the output voltage, revealing consistent performance across batches with spatial variations of 2.53, 4.45, and 4.54 at temperature differences of 20, 40, and 60 K, respectively. This contrasts sharply with traditional devices, where spatial variations exceed 10% even at 20 K temperature differences—over fourfold higher than the integrated device variation at same temperature difference. The observed temporal and spatial variations in thermoelectric devices are also considerably lower than those seen in semiconductor devices, thereby greatly enhancing the reliability of thermoreceptors (Table S2). The low spatial variation of the device can be attributed to the material homogeneity and the narrow *p*–*n* transition region at the interface between thermoelectric materials and metallic connecting materials, achieved through the high-resolution photo-induced patterning of *p*–*n* thermoelectric materials, which mitigates spatial variations in the thermoelectric performance of the device across different locations.

### Nociceptive Behaviors of Thermoelectric Thermoreceptors

**Fig. 3:**
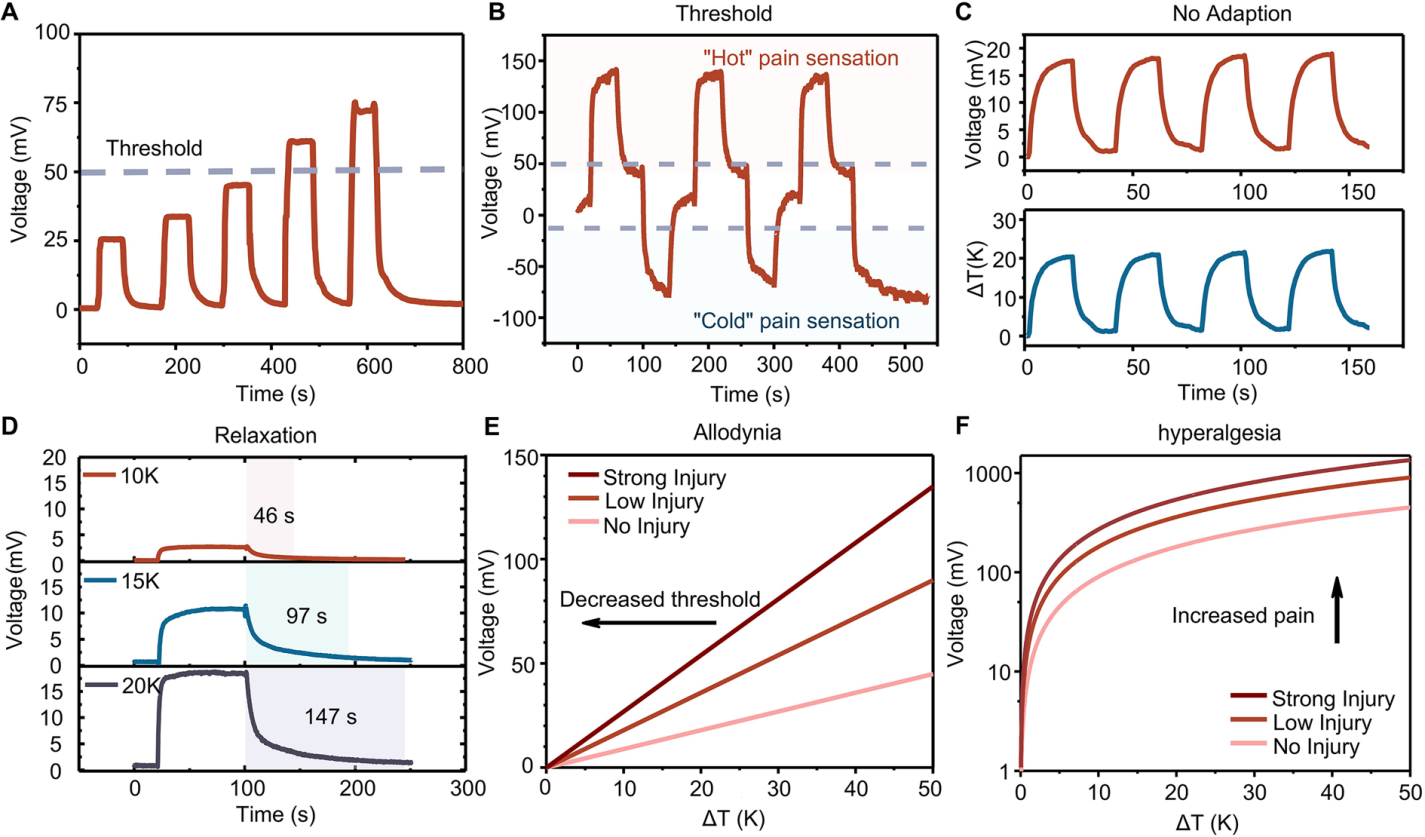
Nociceptive behaviors of artificial thermoreceptor. **A** Threshold behavior: output voltage of the thermoreceptor in response to increasing temperatures. **B** Threshold response to both hot and cold stimuli. **C** No adaptation: output voltage response over repeated pulse duration. **D** Relaxation: relaxation times across different temperature differences. **E** Allodynia hyperalgesia: characteristic responses to normal (no injury, 10 *p*–*n* pair of thermoelectric materials) and abnormal (low injury, 20 *p*–*n* pair of thermoelectric materials and strong injury, 30 *p*–*n* pair of thermoelectric materials) conditions. **F** Hyperalgesia: characteristic responses to both normal (no injury) and abnormal (low injury and strong injury) conditions

Nociceptors are key peripheral sensory neurons that play crucial roles in detecting and avoiding damage from prospective noxious stimuli. The primary advantage of body pain perception lies in its ability to facilitate precise, rapid, and secure interactions with the surrounding environment. Despite the development of numerous devices aimed at mimicking artificial nociceptors, a prevalent challenge among these devices is the presence of inherent temporal and spatial variations, stemming from unregulated ion or electron transport within the medium. These variations compromise the reliability of artificial nociceptors [39]. Due to the exceptional voltage–temperature linearity and the minimized variations of the TX-DBU/CNT *p*–*n* integrated thermoelectric device, the fabricated TX-DBU/CNT *p*–*n* integrated thermoelectric devices have been employed as the sensing component in an artificial thermoreceptor. The thermoelectric devices enable the conversion of thermal energy into electrical signals, effectively addressing the challenge of device power consumption. Nociceptors are characterized by five key features: threshold, non-adaptation, relaxation, allodynia, and hyperalgesia. We simulated the external stimulation of thermoelectric-based self-powered wearable artificial thermoreceptors using heat pulses of varying amplitudes. As shown in Fig. 3A, an increase in stimulus intensity led to a more pronounced output voltage response, observed with both hot and cold stimuli. Figure 3B demonstrates the output voltage of a thermoreceptor in response to these stimuli, reflecting the pain sensation experienced due to extreme temperatures. Interestingly, with constant pulse intensity and duration (Fig. 3C), the output voltage of the thermoelectric-based self-powered wearable artificial thermoreceptors remained consistent, resembling the non-adaptation properties of biological nociceptors. This is a crucial mechanism to protect nociceptors from recurring harmful stimuli.

Relaxation, another important characteristic of nociceptors, was also observed. The output voltage amplitude varied with the interstimulus interval during the relaxation period. When a stimulus of the same pulse duration was applied after complete relaxation, a similar response was generated. However, an enhanced response occurred if the stimulus was applied prior to complete relaxation, indicating that the nociceptor had not fully relaxed (Fig. S6). The correlation between relaxation duration and pulse amplitude was explored, with results shown in Fig. 3D, indicating that relaxation time is positively correlated with the amplitude of the temperature difference. Specifically, relaxation times were measured as 46, 97, and 147 s at temperature differences of 10, 15, and 20 K, respectively. This mirrors the increased relaxation times observed in biological thermoreceptors in response to intense thermal stimuli.

Furthermore, the nociceptor exhibited increased sensitivity in response to excessively intense stimulation, akin to the conditions that cause tissue damage in biological systems. Allodynia and hyperalgesia, indicative of nociceptor injury, were investigated by using thermoelectric-based self-powered wearable artificial thermoreceptors with different *p*–*n* pair to simulate the degree of injury. The results, illustrated in Fig. 3E and F, showed that “injured” thermoreceptors exhibited an increased output voltage and a lowered threshold for a stable output response, reflecting the allodynia and hyperalgesia traits of thermoreceptors.

### Thermo-nociceptive Feedback of Biomimetic Thermo-nociceptive Robotic Arm

**Fig. 4:**
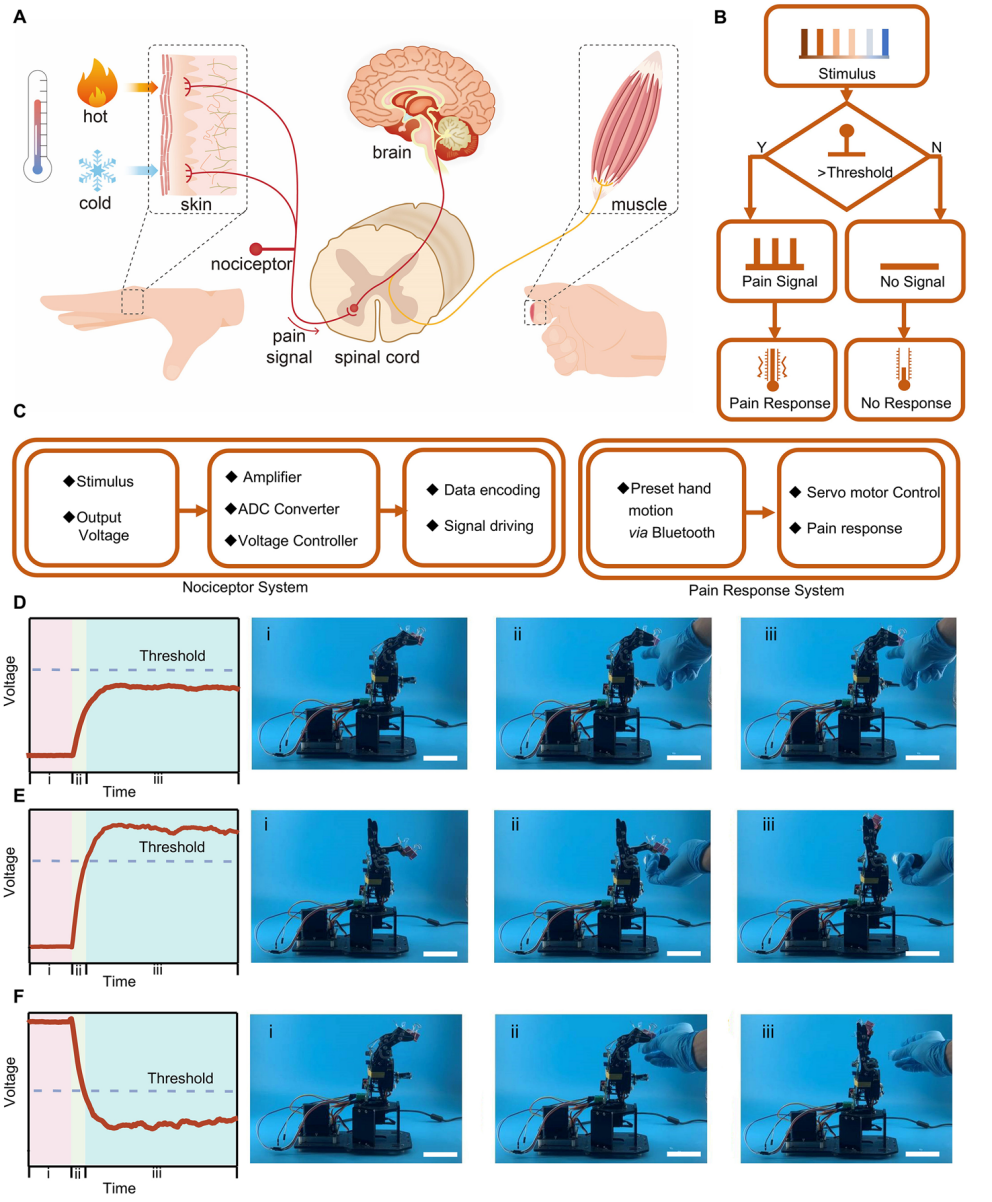
Device structure and mechanism of the biomimetic thermo-nociceptive robotic arm. **A** Schematic of the functional mechanism of a biological nociceptor. **B** Flow diagram detailing the functional mechanism of the artificial thermoreceptor. **C** Device structure of the robotic arm. Pain response of the robotic arm to external thermal stimuli from **D** a human finger (− 309 K), **E** hot water (− 363 K), and **F** an ice block (− 273 K). Scale bar: 5 cm

Building on the impressive simulation capabilities of our thermoelectric-based artificial thermoreceptors to replicate human pain-sensing abilities, we propose an artificial intelligence robotic arm equipped with a pain perception system. This system is intended for further exploration in robotics, prosthetics, and human–computer interaction systems. In humans, thermoreceptors transduce extreme thermal stimuli into long-range electrical signals within the spinal cord, which are then transmitted to higher brain centers. These centers identify the pain area and initiate corresponding reflex commands (Fig. 4A). Similarly, our artificial intelligent robotic arm mimics a human thermoreceptor, generating, transmitting, and executing pain reflex actions in response to extreme thermal stimuli (Fig. 4B, C). Specifically, when the thermoelectric sensing module at the hot end of the thermoelectric material encounters temperature stimulation, it generates a voltage due to the temperature difference. This voltage is then amplified and transmitted to the voltage control module Note S2, Figs. S7–S9). If the voltage exceeds the threshold required to activate the voltage control module, it triggers the servo motor of the robotic arm to execute predetermined actions. Conversely, if the voltage is below this threshold, it fails to activate the servo motor.

Figure 4D–F illustrates specific actions representing the pain response of our artificial robotic arm when subjected to external thermal stimuli. When the robotic arm comes into contact with a human finger at approximately 309 K, it categorizes the interaction as a begin touch and does not respond. Nevertheless, the robotic pain reflexes are triggered by harmful touches from either hot water (− 363 K) or ice blocks (− 273 K). When hot water approaches the tip of the robotic arm’s index finger, the finger quickly lifts to avoid potential harm. Similarly, when ice blocks approach, the palm of the robotic arm swiftly opens to prevent potential damage.

In the forthcoming era of robotics, where human–robot interactions in dynamic environments will become common, the precise perception of the external world and subsequent adaptive behavior are essential for robots. Integrating the capacity to discern pain levels in robots is crucial for enhancing their self-protection and minimizing human casualties. Given their susceptibility to mishaps during tasks, such as exposure to high temperatures, artificial thermoelectric-based self-powered wearable artificial thermoreceptors are expected to play a key role. These devices enable robots to detect pain levels and initiate appropriate protective responses, reducing the likelihood of accidents.

**Fig. 5:**
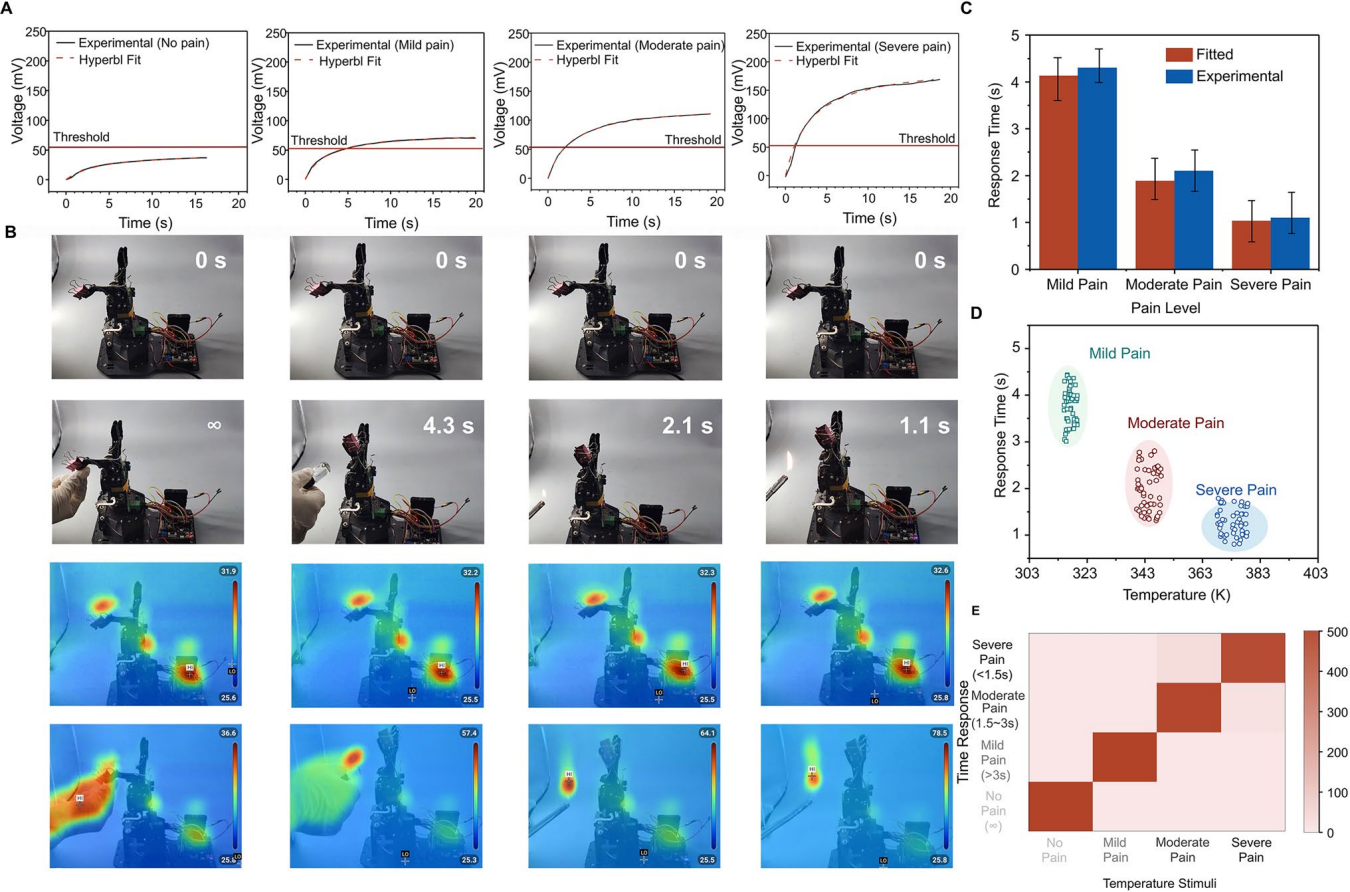
Thermo-nociceptive protective response behavior of the biomimetic thermo-nociceptive robotic arm. **A** Voltage variation over time in response to different pain levels, presented experimentally and with Hyperbl fit. **B** Photographs and infrared images of the robotic arm’s pain response to different thermal stimuli. Scale bar: 2 cm. **C** Hyperbl fitted and experimental data on the response time of the artificial thermoreceptor at different pain levels. **D** Response time of the robotic arm under varying temperatures. **E** Confusion matrix displaying classification accuracy of pain levels by analyzing thermal stimuli and response time

The voltage generated by the thermoelectric-based artificial thermoreceptors varies according to the severity of the pain conditions—mild, moderate, and severe—facilitating the operation of the robotic arm. Conversely, in no-pain conditions, the voltage does not surpass the threshold, preventing unintended activation. The voltage response is modeled using the Hyperbl fit function, as visualized in Figs. 5A and S10, S11. The fitted data aligns well with our experimental observations (Fig. 5C), showing that the robotic arm produces a voltage that correlates positively with temperature changes, leading to varied response times. This capability allows for simulating different pain levels induced by thermal stimuli and dynamically adjusting the response time accordingly.

As depicted in Fig. 5B, the thermo-nociceptive feedback of the robotic arm with a pain perception system categorizes pain into four distinct levels, corresponding to human physiological responses to thermal stimuli: no pain (< 318 K), mild pain (318–333 K), moderate pain (333–348 K) and severe pain (> 348 K). The time needed to reach the pain threshold decreases as the pain level increases. At temperatures below 318 K, the robotic arm remains at the no-pain level without generating protective feedback. However, once the temperature exceeds the critical threshold of 318 K, the robotic arm registers pain and provides protective feedback regarding the elevated temperature. Furthermore, the robotic arm exhibited responses indicative of abnormal pain mechanisms. As demonstrated in Fig. S13, devices integrated with 20 *p*–*n* thermoelectric pairs (simulating low injury) and 30 *p*–*n* thermoelectric pairs (simulating strong injury) revealed distinct nociceptive characteristics when subjected to identical thermal stimuli. The injured robotic arm showed lower response time under thermal stimuli compared to no-injury robotic arm, effectively mimicking allodynia and hyperalgesia observed in biological thermoreceptors.

There is a positive correlation between increasing temperature and heightened pain levels, resulting in shorter reaction times for providing protective feedback. Specifically, as the robotic arm progresses through mild, moderate, and severe pain levels, the corresponding reaction times are 4.3, 2.1, and 1.1 s, respectively (Movies S1, S2, S3). To assess the accuracy of the robotic arm’s pain-level perception under static large-sample stimuli, we constructed a confusion matrix (Fig. 5D, E). With a sample size of 500 in each category, the classification accuracy exceeded 98.6%, indicating the thermoreceptor system’s exceptional capability to accurately perceive and respond to potentially harmful thermal stimuli.

## Conclusions

In this study, we have innovatively developed an air-stable photo-induced *n*-type dopant, along with a sophisticated photo-induced patterning technology, to construct high-precision joint-free *p*–*n* integrated thermoelectric devices. Notably, the exceptional stability of the photobase generator post-UV exposure, coupled with our meticulously engineered joint-free device architecture, has resulted in remarkable performance characteristics. Specifically, the device exhibited extremely low temporal variations of less than 1% and spatial variations of 4.54%. These minimized variations, coupled with superior linearity, position our devices as viable candidates for artificial thermoreceptors capable of sensing external thermal noxious stimuli. Our artificial thermoreceptors successfully replicated key nociceptor features, including threshold, non-adaptation, relaxation, allodynia, and hyperalgesia. This replication underscores the fidelity and robustness of our approach. Furthermore, by integrating these artificial thermoreceptors with a robotic arm, we demonstrated the effectiveness of our innovation in generating pain responses in the robotic arm when subjected to external thermal stimuli. This system not only discerned pain levels accurately but also initiated appropriate protective responses across a spectrum of intensities. Our findings offer a novel strategy for the construction of high-resolution thermoelectric sensing devices, paving the way for the development of precise biomimetic thermoreceptors. These advancements have the potential to significantly impact the fields of robotics and prosthetics.

## Supplementary Information

Below is the link to the electronic supplementary material.

Supplementary file1 (MP4 7093 KB)

Supplementary file2 (MP4 5837 KB)

Supplementary file3 (MP4 8929 KB)

Supplementary file4 (DOCX 1979 KB)
