# Supplementary material for: High-Reliability Thermoreceptors with Minimal Temporal and Spatial Variations Through Photo-Induced Patterning Thermoelectrics
Source: Nanomicro Lett. 2025 Jun 23;17:307. doi: 10.1007/s40820-025-01821-1 (PMC12185834; doi:10.1007/s40820-025-01821-1)
Supplement: Supplementary file 4 — Supplementary file4 (DOCX 1979 KB) [file 40820_2025_1821_MOESM4_ESM.docx]

Supporting Information for

**High-Reliability Thermoreceptors with Minimal Temporal and Spatial Variations through Photo-Induced Patterning Thermoelectrics**

Chunyu Du^1^, Yue Hu^1^, Xiao Xiao^2^, Farid Manshaii^2^, Lirong Liang^1^, Jun Chen^2,3,^* and Guangming Chen^1^*

^1^ College of Materials Science and Engineering, Shenzhen University, Shenzhen 518055, P. R. China

^2^ Department of Bioengineering, University of California, Los Angeles, Los Angeles, CA 90095, USA

^3^ SKKU Institute of Energy Science and Technology, Sungkyunkwan University, Suwon 16419, Republic of Korea

*Corresponding authors. E-mail: [chengm@szu.edu.cn](mailto:chengm@szu.edu.cn) (Guangming Chen); [junucla@ucla.edu](mailto:junucla@ucla.edu) (Jun Chen)

Note S1 Artificial thermoelectric nociceptor and biological nociceptor

Artificial thermoelectric nociceptors closely resemble biological nociceptors in several aspects (**Fig. S1**): (1) they share a sensing mechanism that translates harmful external thermal stimuli into electrical signals to activate protective mechanisms. (2) Both human skin and artificial devices share a comparable three-level structural composition, encompassing protection, sensing, and transmission. Specifically, the artificial thermoelectric nociceptor and biological nociceptor reside within the same layer known as the sensing layer.

Futhermore thermoelectric thermoreceptors offer three additional advantages over conventional CMOS, transistor, or resistive memory nociceptors. (1) The thermoelectric thermoreceptors operate based on the Seebeck effect, where the thermoelectric potential directly correlates with the temperature difference across the device. Thus, device variations are effectively minimized. (2) Thermoelectric thermoreceptors possess a simple device structure that enables integration. (3) Thermoelectric thermoreceptors can effectively address the endurance issue by utilizing heat sources for direct power supply.

Note S2 Response time of the biomimetic thermo-nociceptive robotic arm with different pain level

The temperature perception of thermoreceptor is instantaneous. Upon receiving intense thermal stimulation, the hot end of the thermoreceptor rapidly approximates the temperature of the heat source, while the cold end maintains the ambient temperature. The temperature of thermoelectric thermoreceptor for generating pain is set to 85 °C, and the ambient temperature is around 25 °C. Thus, the temperature difference is estimated to 60 °C (60 K). The thermoelectric thermoreceptor exhibits an open-circuit voltage of 50.8 mV at a temperature difference of 60 K. In other words, when the voltage surpasses 50.8 mV, the amplified output power from the voltage amplifier exceeds the power of the voltage control module, enabling the operation of the robotic arm. Therefore, the minimum voltage generated by thermoelectric thermoreceptor to drive the robotic arm is 50.8 mV.

The voltage generated by the thermoelectric nociceptor is fitted using the Hyperbl fit function in Origin Software. The fitted data shows a good fit, and the response time calculated using the Hyperbl fit equation closely matches our experimental data (**Figs. S10-S11**). This indicates that the robotic arm can produce a voltage that correlates positively with different temperatures, resulting in a corresponding decrease in reaction time. This allows for the simulation of different pain levels induced by varied thermal stimuli, with the added feature of dynamically adjusting the response time.

S3 Supplementary Figures


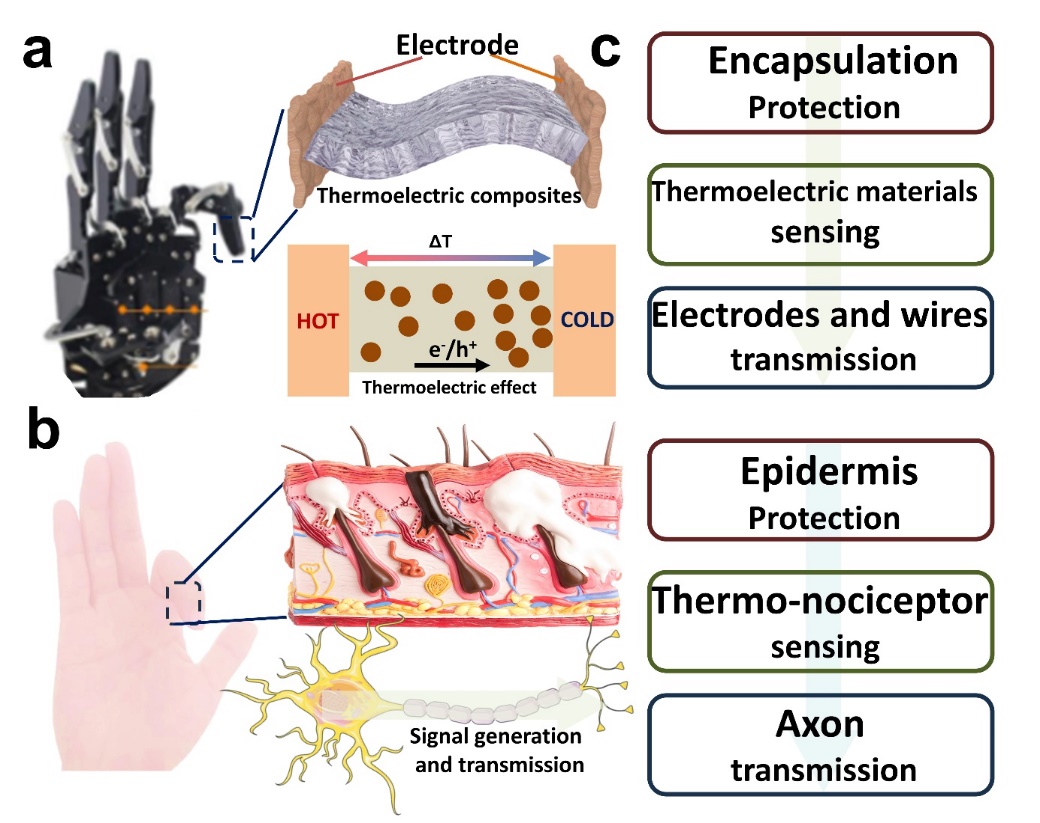


**Fig. S1 a**) Artificial thermoelectric nociceptor: thermoelectric nociceptive robotic arm (left), device structure (middle top) and mechanism of thermoelectric effect (middle bottom); **b**) biological nociceptor: human hand (left), nociceptive sensory system structure (middle top) and mechanism of signal generation and transmission (middle bottom); and c) the comparison of their structure and function


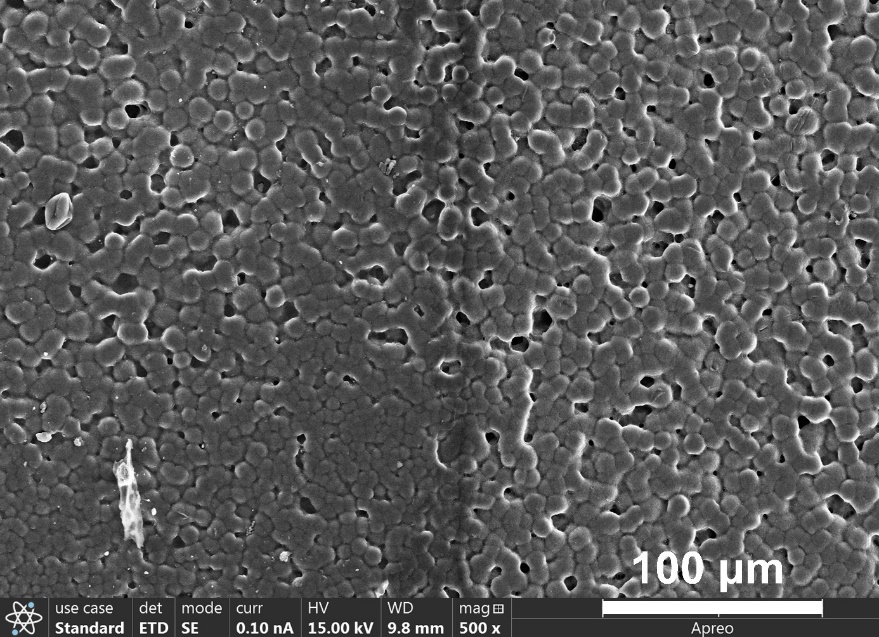


**Fig. S2** SEM of the transition of p-n integrated thermoelectric composite





**Fig. S3** Comparison of temporal and spatial variation between traditional device and integrated device


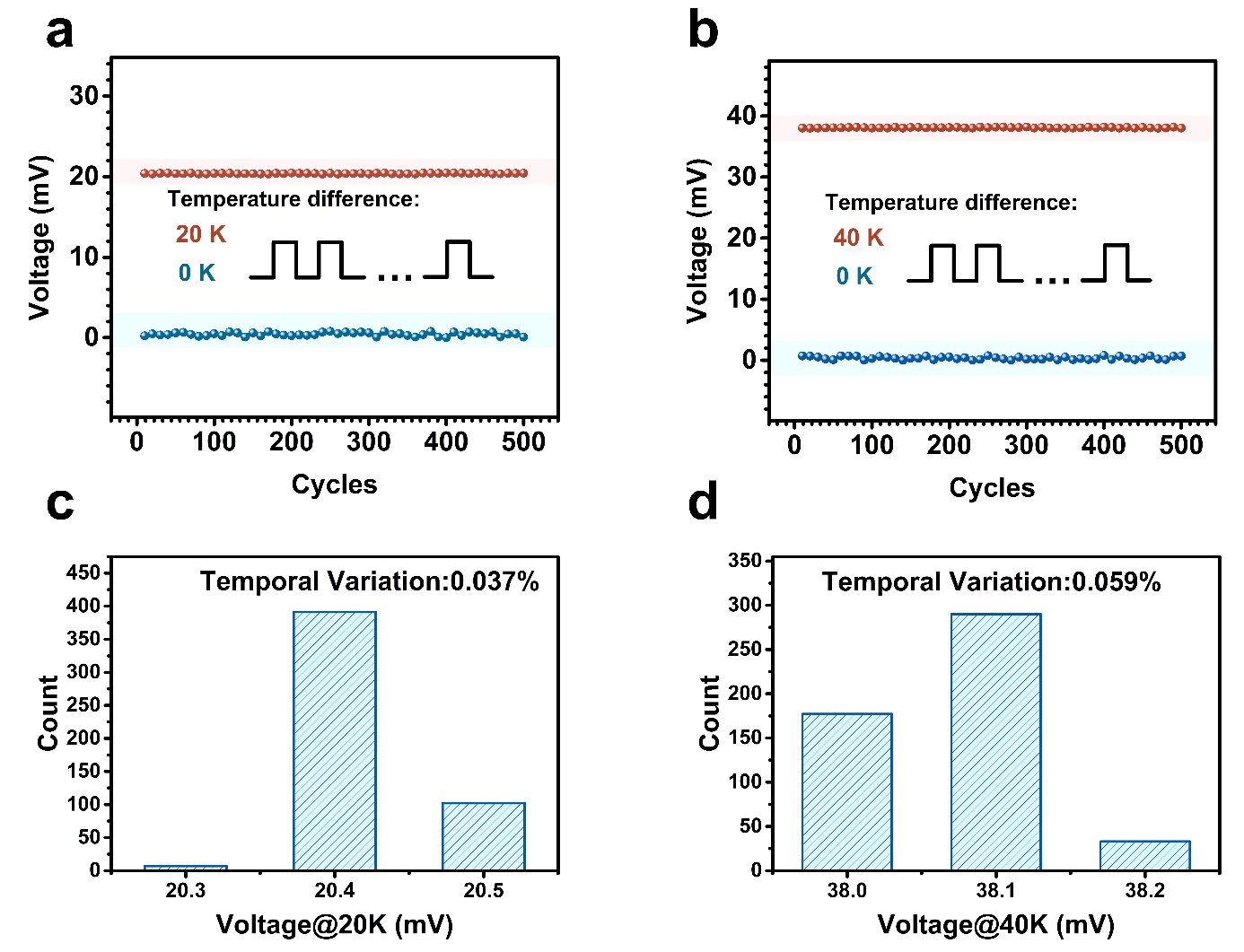


**Fig. S4** The temporal variation with temperature difference at (**a**) 20K and (**b**) 40 K. The histogram of the output voltage distribution with temperature difference at (**c**) 20K and (**d**) 40 K





**Fig. S5** Voltage stability of the integrated thermoelectric device under repeated bending cycles





**Fig. S6** The output voltage of the thermoelectric thermoreceptor is observed to remain constant after attaining a saturated intensity





**Fig. S7** Open-circuit voltage stability of the artificial thermoreceptor in 30-day ambient condition





**Fig. S8** Power stability of the artificial thermoreceptor in 30-day ambient condition with an loading resistance of 0.8 Ω


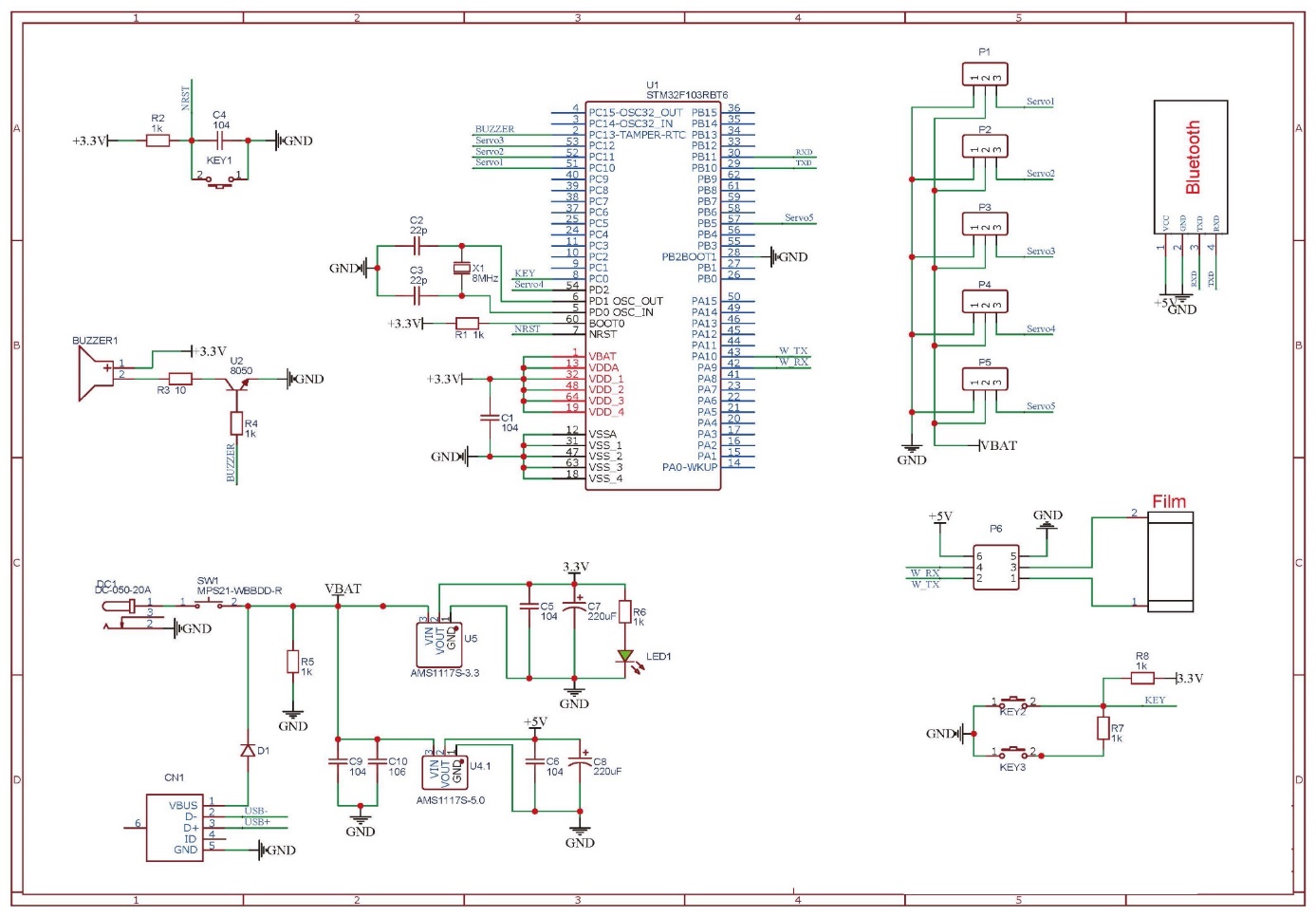


**Fig. S9** Circuit diagram of biomimetic thermo-nociceptive robotic arms


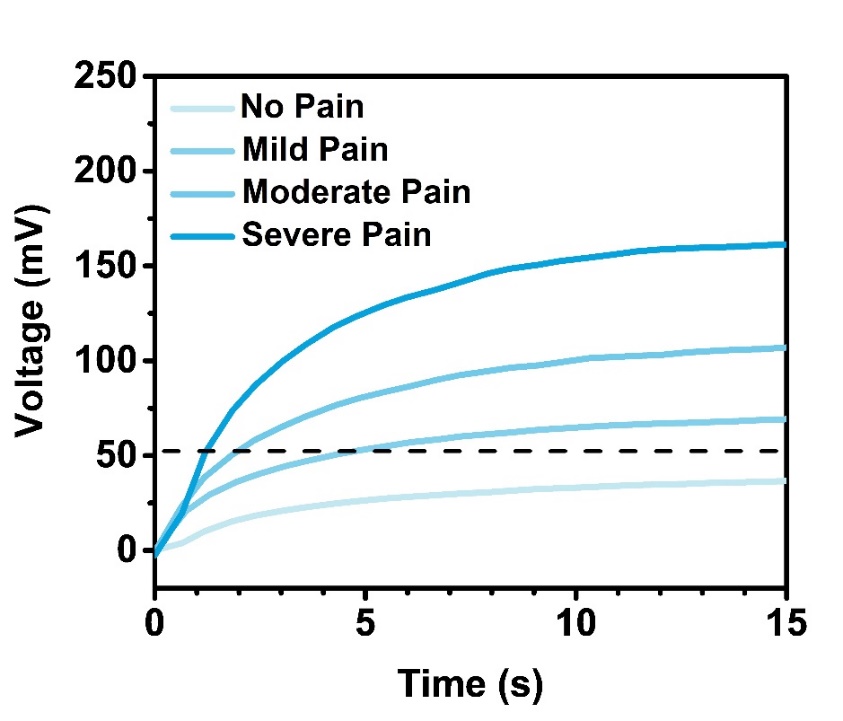


**Fig. S10** Voltage as a function of time with different pain level


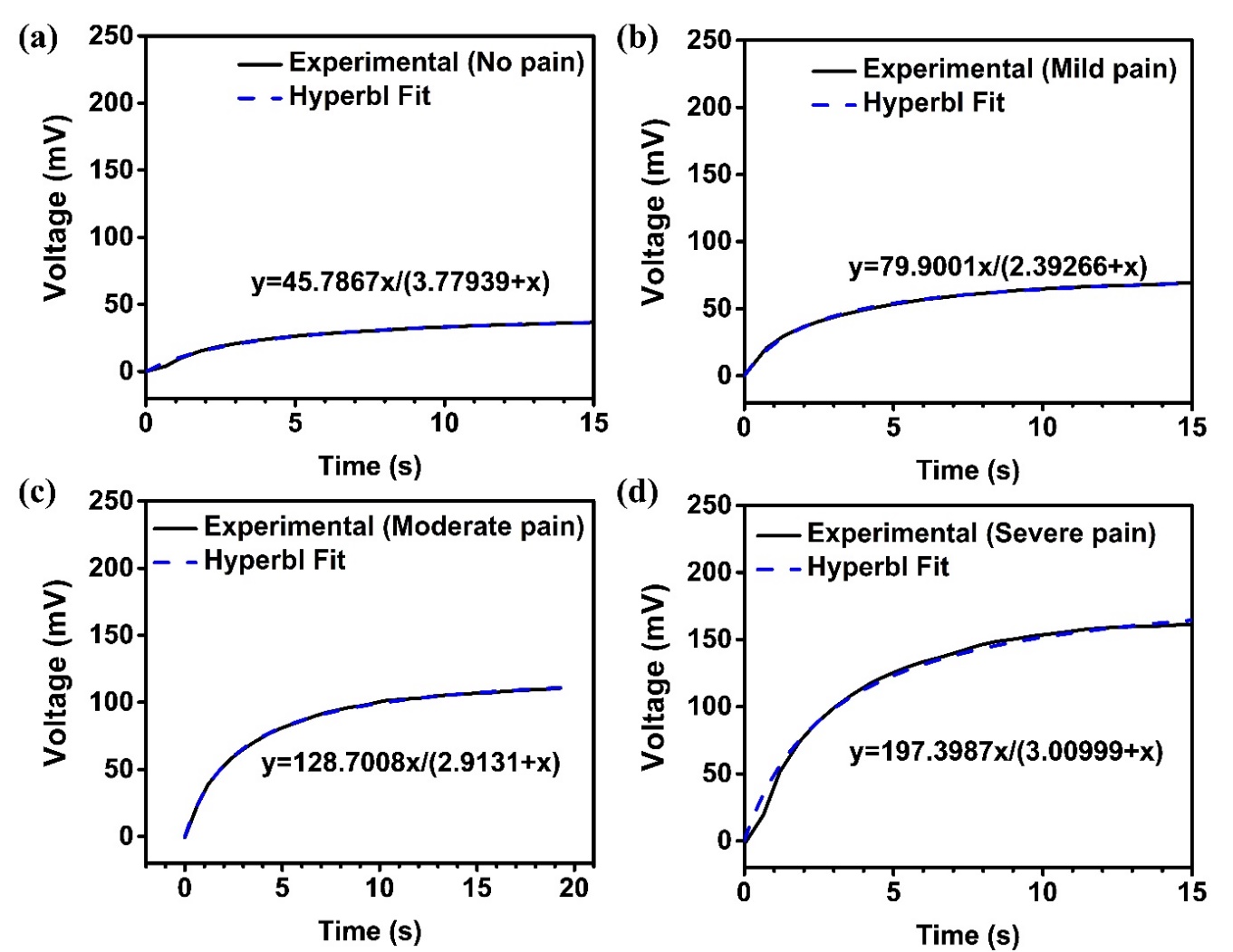


**Fig. S11** Voltage as a function of time with different pain level with different pain level in the experiment and Hyperbl fit





**Fig. S12** Response time of non-injury and injury robotic arm

Table S1 Comparison of Artificial thermoelectric nociceptor and biological nociceptor

|  | Mechanism | Structure | Basic Functionality | Advanced Functionality |
| --- | --- | --- | --- | --- |
| Artificial thermoelectric thermoreceptor | Translating overheated thermal stimuli into electrical signals to drive the device | Sensing layer in skin | “threshold”,  “no adaption”, “relaxation”, “allodynia” and “hyperalgesia | Signal corresponding to different pain level generated by different stimuli in static and dynamic conditions |
| Biological nociceptor | Translating harmful external stimuli into electrical signals to activate protective mechanisms | Sensing layer in sensors | “threshold”,  “no adaption”, “relaxation”, “allodynia” and “hyperalgesia | Signal corresponding to different pain level generated by different stimuli in static and dynamic conditions |

Table S2 Variation comparison of thermoelectric thermoreceptor and semiconductor nociceptor

| Device | Temporal Variation | Spatial Variation | Refs. |
| --- | --- | --- | --- |
| Crystalline-SiGe-based RRAM nociceptor | 1% | 4.9% | [S1] |
| ZnO-based RRAM nociceptor | 10% | 25% | [S2] |
| NiO/SiO_X_-based RRAM nociceptor | 11% | 30% | [S3] |
| SiO_2_-based RRAM nociceptor | 32% | 34% | [S4] |
| BN-based RRAM/TENG nociceptor | >5% | ~22% | [S5] |
| Thermoelectric thermoreceptor | <1% | <5% | This work |

Table S3 Overall performance Comparison of thermoelectric thermoreceptor and semiconductor nociceptor

| Device | Flexibility | Self-powered | Variation | Price | Ref. |
| --- | --- | --- | --- | --- | --- |
| RRAM nociceptor | √(Flexible substrate)  ×(Inflexible substrate) | × | Temporal:1-35%  Spatial:5-45% | >$1 | S1-S3 |
| RRAM nociceptor with self-powered device | × | √ | Temporal:>5%  Spatial:~20% | >$5 | S4-S5 |
| Thermoelectric thermoreceptor | √ | √  (Sensing Unit) | Temporal:<1%  Spatial:<5% | <$0.5 | This work |

Movie S1 Nociceptive behavior of the biomimetic thermo-nociceptive robotic arm under mild-level pain stimuli

Movie S2 Nociceptive behavior of the biomimetic thermo-nociceptive robotic arm under moderate-level pain stimuli

Movie S3 Nociceptive behavior of the biomimetic thermo-nociceptive robotic arm under severe-level pain stimuli

Supplementary References

1. S. Choi, S.H. Tan, Z. Li, Y. Kim, C. Choi et al., *SiGe epitaxial* memory for neuromorphic computing with reproducible high performance based on engineered dislocations. Nat. Mater. **17**(4), 335–340 (2018). <https://doi.org/10.1038/s41563-017-0001-5>
2. J.H. Park, D.S. Jeon, T.G. Kim, Improved uniformity in the switching characteristics of ZnO-based memristors using Ti sub-oxide layers. J. Phys. D Appl. Phys. **50**(1), 015104 (2017). <https://doi.org/10.1088/1361-6463/50/1/015104>
3. B.K. You, W.I. Park, J.M. Kim, K.I. Park, H.K. Seo et al., Reliable control of filament formation in resistive memories by self-assembled nanoinsulators derived from a block copolymer. ACS Nano **8**(9), 9492–9502 (2014). <https://doi.org/10.1021/nn503713f>
4. Y.-C. Huang, W.-L. Tsai, C.-H. Chou, C.-Y. Wan, C. Hsiao et al., High-performance programmable metallization cell memory with the pyramid-structured electrode. IEEE Electron Device Lett. **34**(10), 1244–1246 (2013). <https://doi.org/10.1109/LED.2013.2275851>
5. G. Ding, R.-S. Chen, P. Xie, B. Yang, G. Shang et al., Filament engineering of two-dimensional h-BN for a self-power mechano-nociceptor system. Small **18**(16), 2200185 (2022). <https://doi.org/10.1002/smll.202200185>
